# An Inquiry

**Published:** 1894-01

**Authors:** 


### An Inquiry.

The following inquiry has just come to hand, which we here with give, with a remark or two, hoping that it may draw out the experience of others on the points here suggested. The inquiry is as follows:

"Have you ever met with serious disturbances following operations upon the teeth or the wearing of badly-fitting plates—such disturbances, for instance, as facial spasm, or lingual spasm, or masticatory spasm? Have you read of such anywhere? I should be greatly obliged for an answer. F. P."

In reply to the above inquiry we may say, that affections of either an inflammatory or neuralgic character may attend or follow improper or unskillful operations upon the natural teeth, as has been witnessed in many instances. It is frequently the case that inflammatory conditions are occasioned by ill-fitting plates; neuralgic affections do not often occur from this cause, but sometimes do.

In a recent case in which a continuous gum denture for a lower jaw was inserted, though no abrasion or inflammation was anywhere apparent, yet when the piece was placed in the mouth, though to all appearances a well-fitted plate, there was severe pain after it had been worn a short time, so that its use had to be abandoned, and return to the use of the old plate, which was much less perfect in its adaptation.

We have known a few cases of this kind, they do not, however, often occur.

Inflammation of the mucous membrane and soft parts of the mouth is of very frequent occurrence from ill-fitting plates; usually this occasions complaint and remedy, but in many instances the irritation comes on so gradually and almost imperceptibly as to inure the patient to the inconvenience till now and again serious conditions are established.

Another inconvenience that is experienced by this wearing ill-fitting plates, is defective mastication and insalivation, and a corresponding inefficiency in the digestive and nutrient processes.

This is a subject to which far more attention should be given by our profession than it has hitherto had. We trust this suggestion will elicit such attention as will cause every such case to be investigated and studied.

